# Insights into Sorption–Mineralization Mechanism for Sustainable Granular Composite of MgO-CaO-Al_2_O_3_-SiO_2_-CO_2_ Based on Nanosized Adsorption Centers and Its Effect on Aqueous Cu(II) Removal

**DOI:** 10.3390/nano12010116

**Published:** 2021-12-30

**Authors:** Alla G. Morozova, Tatiana M. Lonzinger, Vadim A. Skotnikov, Gennady G. Mikhailov, Yury Kapelyushin, Mayeen Uddin Khandaker, Amal Alqahtani, D. A. Bradley, M. I. Sayyed, Daria I. Tishkevich, Denis A. Vinnik, Alex V. Trukhanov

**Affiliations:** 1 Laboratory of Single Crystal Growth, South Ural State University, 454080 Chelyabinsk, Russia; morozovaag@susu.ru (A.G.M.); lonzingertm@susu.ru (T.M.L.); skotnikovva@susu.ru (V.A.S.); mikhailovgg@susu.ru (G.G.M.); kapeliushinye@susu.ru (Y.K.); dashachushkova@gmail.com (D.I.T.); denisvinnik@gmail.com (D.A.V.); 2 Centre for Applied Physics and Radiation Technologies, School of Engineering and Technology, Sunway University, Petaling Jaya 47500, Selangor, Malaysia; mayeenk@sunway.edu.my (M.U.K.); d.a.bradley@surrey.ac.uk (D.A.B.); 3 Department of Basic Sciences, Deanship of Preparatory Year and Supporting Studies, Imam Abdulrahman Bin Faisal University, Dammam 34212, Saudi Arabia; amalqahtani@iau.edu.sa; 4 Centre for Nuclear and Radiation Physics, Department of Physics, University of Surrey, Guildford GU2 7XH, UK; 5 Department of Physics, Faculty of Science, Isra University, Amman 11622, Jordan; dr.mabualssayed@gmail.com; 6 Department of Nuclear Medicine Research, Institute for Research and Medical Consultations (IRMC), Imam Abdulrahman Bin Faisal University, Dammam 31441, Saudi Arabia; 7 Laboratory of Magnetic Films Physics, SSPA “Scientific-Practical Materials Research Centre of NAS of Belarus”, 220072 Minsk, Belarus

**Keywords:** sorption mechanism, nanosized adsorption centers, copper pollution, mineralization, irreversible sorbent, sustainable

## Abstract

Although copper is needed for living organisms at low concentrations, it is one of the pollutants that should be monitored along with other heavy metals. A novel and sustainable composite mineralizing sorbent based on MgO-CaO-Al_2_O_3_-SiO_2_-CO_2_ with nanosized adsorption centers was synthesized using natural calcium–magnesium carbonates and clay aluminosilicates for copper sorption. An organometallic modifier was added as a temporary binder and a source of inovalent ions participating in the reactions of defect formation and activated sintering. The sorbent-mineralizer samples of specified composition and properties showed irreversible sorption of Cu^2+^ ions by the ion exchange reactions Ca^2+^ ↔ Cu^2+^ and Mg^2+^ ↔ Cu^2+^. The topochemical reactions of the ion exchange 2OH^−^ → CO_3_^2−^, 2OH^−^ → SO_4_^2−^ and CO_3_^2−^ → SO_4_^2−^ occurred at the surface with formation of the mixed calcium–copper carbonates and sulfates structurally connected with aluminosilicate matrix. The reverse migration of ions to the environment is blocked by the subsequent mineralization of the newly formed interconnected aluminosilicate and carbonate structures.

## 1. Introduction

Heavy metals are major pollutants that must be monitored in all countries [1]. The source of pollution in water bodies is wastewater [2] from industrial enterprises of non-ferrous and ferrous metallurgy, mining, machine building and chemical and automobile [3] industries. The heavy metal elements contaminate the soil and water bodies near the sites of mining and manufacturing. Natural barriers that can block contaminants are zeolites, clay minerals [4], carbonate rocks [5], magnesium hydroxides [6] and zeolitic tuff [7]. However, natural barriers do not cope with the scale of anthropogenic pollution, and the migration of ions to liquid media is not limited by transport difficulties. Absence of natural or synthetic barriers leads to contamination of the biosphere over the vast areas by heavy metal elements [8], which stimulates research and development of techniques to cope with anthropogenic pollution. One of the elements monitored by environmentalists, along with other pollutants, is copper [9]. Although copper is needed for living organisms at low concentrations and the human body can regulate its level, at high concentrations it is toxic and may even be fatal. The epidemiological evidence, such as higher cancer incidence around the copper production sites, has been confirmed [10]. There is evidence of stomach and intestinal problems in the human body copper levels above 1.3 mg L^−1^ for even short exposure periods. Longer exposure may even cause kidney and liver damage [11]. At high concentrations, copper is used to control algal blooms. In addition, there can be harmful effects on zooplankton [12], fish and invertebrates [13]. 

The developed approaches to remove copper include chemical precipitation [14,15], electrochemical treatments [16,17], adsorption [18,19,20,21,22], ion exchange [23] and membrane separation [24]. It is known to use natural inorganic aluminosilicate materials and complex organic polymeric systems with specially formed, selective sorption with respect to certain substances as sorbents [25]. The disadvantage of natural materials is the reversibility of sorption processes and the possibility of a burst release of sorbed components. The synthesis of polymer systems is currently expensive, which hinders their use. Furthermore, complex phosphates attract great attention of the researchers [26]. There is a number of studies on sorption of copper ions by organic ions, clays, zeolites, polysiloxanes and cellulose. In these studies, the concentration of copper ions in sorbate solution was low (5–50 mg/L). The adsorption was in the range of 0.25–0.5 mmol/L and the maximum sorption capacity did not exceed 3 mg/g [27,28,29,30,31].

The most simple, effective and economic method is sorption. However, the problem of limited sorbent intake and the reversibility of the sorption process stimulate deeper research to design novel methods. In order to sorb and retain copper irreversibly, one potential source material is metallurgical slag. Millions of tons of slag dumps have been accumulated as a result of metal production by metallurgical enterprises. Slag dumps are technogenic deposits of silicate materials with a wide range of potential applications. The chemical, phase and dispersion composition of metallurgical slags depend on their chemical and thermal prehistory during smelting of metals. The main components of metallurgical slags are usually mixed oxides of the CaO-Al_2_O_3_-SiO_2_ system. In our previous work, the ability of a fine-crumbling metallurgical slag to perform irreversible sorption of Ce^3+^ ions from solution was observed and investigated [32]. However, the mechanism of irreversible sorption by slag-based sorbent was not studied in detail. The chemical composition of the suitable slag was quite untypical, and not every metallurgical company in the world produces it. A characteristic feature of the sorbent obtained from the slag was the formation of a mechanically and chemically stable aluminosilicate framework [33,34]. In fact, the technology of the composite sorbent based on metallurgical slag was two-stage: at the first stage, the initial phase components were synthesized by the metallurgical enterprise as a result of the silicate decomposition of slag after metal smelting, and during the second stage, the slag decomposition products were used for obtaining the composite granular sorbent. Issues with very specific metallurgical slag composition stimulate research to identify other raw material sources suitable for the sorbent obtaining that would significantly widen the sorbent applicability.

Thereby, the objectives of present work were to: synthesize a mineralizing composite sorbent based on the MgO-CaO-Al_2_O_3_-SiO_2_-CO_2_ system from other materials, avoiding metallurgical slag (i.e., natural calcium and magnesium carbonates, aluminosilicate clay materials and organometallic compound based on esters, as a modifier); improve sorption irreversibility by the introduction of carbonates to raw material; investigate Cu^2+^ sorption capacity; and provide insights into the sorption–mineralization mechanism of this system.

The sorption–mineralization capability of a newly obtained sorbent based on nanosized adsorption centers during interaction with a sorbate solution is considered as the capability to form new water-insoluble formations on the surface, which are structurally connected with the aluminosilicate matrix that would exclude the migration of the Cu^2+^ ions to the environment. In comparison with the previously obtained sorbent from metallurgical slag, the newly synthesized one contains carbonates. Another characteristic feature is the application of organometallic modifier, which is simultaneously used as a temporary binder and a source of inovalent ions. The advantage of using the new adsorbent in comparison with others is the ability to reduce the content of carbon dioxide in the atmosphere, due to the formation of carbonates on the surface of the adsorbent after the end of the sorption process. The quantity of carbonates is equal to 4%–5% by weight of the adsorbent.

## 2. Materials and Methods

### 2.1. Preparation of Sorbent

The composite sorbent was synthesized based on the MgO-CaO-Al_2_O_3_-SiO_2_-CO_2_ system. The following components were used as reagents for sorbent synthesis: natural calcium and magnesium carbonates (dolomite); natural aluminosilicate clay materials (bentonite); and organometallic modifier (cellulose glycolic acid salt). The ratio of reagents was chosen based on formation of the phase components, which were isostructural to helenite Ca_2_Al[AlSiO_7_] and sennonite ɣ-Ca_2_[SiO_4_] in the composition of sorbent during further sintering (the ratio of elements Ca, Mg, Al, Si was 2:1:1:3). It was expected that the use of calcium–magnesium carbonate as an active component of the solid phase synthesis might strengthen the role of Mg^2+^ ions in the ion exchange reaction between sorbent and sorbate. The initial mixed calcium–magnesium carbonate was used with particle size in the range of 1 to 250 μm. In comparison with the slag-based sorbent [32], an organometallic compound based on esters was added as a modifier. The chemical composition of the obtained mixture is provided in Table 1.

**Figure 1:**
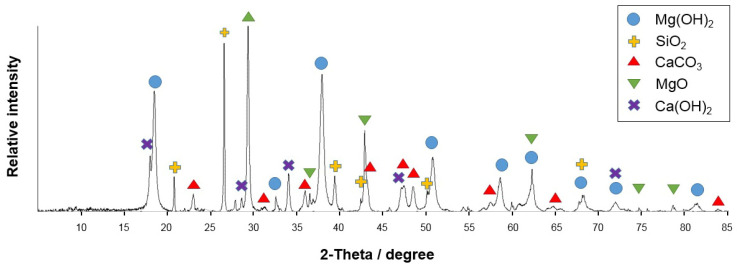
XRD analysis of initial mixture.

The prepared mixture was analyzed by the XRD diffraction analysis; the phase composition is shown in Figure 1.

According to the XRD analysis, the major phase of the prepared mixture was magnesium hydroxide Mg(OH)_2_. No aluminum-containing phase was detected; this could be associated with the amorphous state of the aluminum-containing phase.

**Figure 2:**
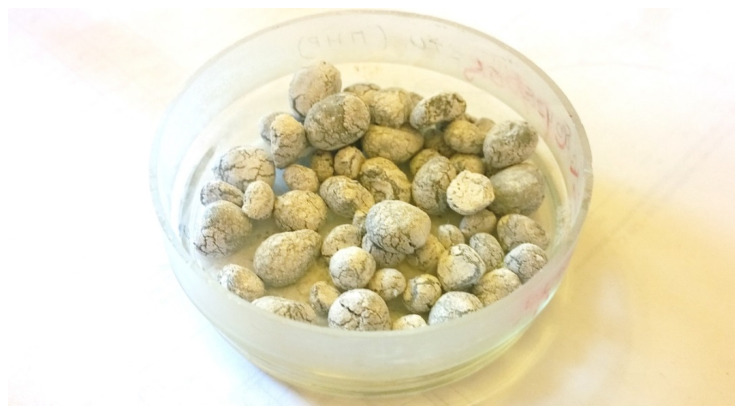
Granules of unsintered sorbent.

The mixture was granulated for obtaining 1–10 mm granules. The obtained samples of sorbent in the form of granules are presented in Figure 2.

### 2.2. Sintering

Sintering enables extension of the possibilities for directional regulation of structure, and the properties of sorbent and its effects on further processes of sorption and chemical reactions. The sintering operation was guided by the known method of activating the reactivity of solids (Hedvall effect [35,36]), i.e., inovalent alloying with Na^+^ and K^+^ ions, which activate the defect-formation reactions and reactive diffusion of ions [37]. The temperature range from 850 to 1350 °C was chosen based on Tammann temperature calculation (T_sint_ = 0.7T_melt_) [38,39]. Thereby, the thermal treatment of the granules was performed at this temperature.

### 2.3. Experimental Procedure

#### 2.3.1. Sorption

The samples synthesized at different temperatures were tested for sorption capacity during interaction with copper sulfate solution. Further investigation of the sorption process and reactivity of the sintered granules was performed by the limited volume method under static conditions with a sorbent/sorbate mass ratio of 1:30 (33.333 g of sorbent was introduced into 1 L of solution). A model solution of copper sulfate with a concentration of 370 mg/L and pH of 3.8 was used as sorbate. The sorption–mineralization experiments were performed under static conditions for 7 days.

#### 2.3.2. Desorption

The sorbent granules, after contact with the model solution, were placed in distilled water with a sorbent/sorbate mass ratio of 1:20. After 30 days, the water composition was analyzed. The concentrations of elements in the solution were determined using an atomic emission spectrometer.

### 2.4. Sorbent Characterization

The initial, sintered and reacted samples of the sorbent granules were studied at ambient temperature by XRD diffraction analysis using XRD diffractometer Rigaku Ultima IV with software “SiroQuant” (Wilmington, NC, USA), allowing quantitative phase analysis; by infrared spectroscopy with the Netzsch STA 449C Jupiter with mass spectrometer, Aeolos II: Netzsch (Selb, Germany); by electron microscope analysis with JEOL JSM 6460 LV (“JEOL Ltd.”, Tokyo, Japan); and by chemical analysis with atomic emission spectrometer equipped with inductively coupled plasma Optima 2100DV (“PerkinElmer Inc.”, Waltham, MA, USA).

## 3. Results and Discussion

### 3.1. Sorption of Cu^2+^ Ions

**Figure 3:**
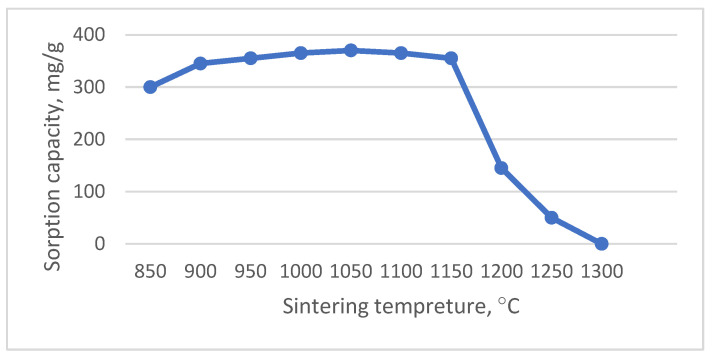
Effect of sintering temperature on sorption capacity of Cu^2+^ ions from copper sulfate solution by sorbent.

The effects of sintering temperature of the sorbent samples on sorption capacity of Cu^2+^ ions are provided in Figure 3.

As shown in Figure 3, the three critical temperature intervals are distinguished, which could be associated with the features of phase formation during synthesis of a sorbent, and the nature of interaction in the sorbent/sorbate system:

I—formation of a fine-dispersed precipitate in the solution volume, accompanied by a decrease in concentration of Cu^2+^ ions in sorbate solution of between 93% and 95% (from 850 to 1000 °C);

II—decrease in concentration of Cu^2+^ ions of between 92% and 95%, accompanied by the absence of precipitate in the solution volume and an increase in sorbent mass by 0.6 wt.%, (from 1000 to 1150 °C);

III—deactivation of the sorbent surface and reduction in Cu^2+^ ions in the sorbate solution by ~20% (from 1200 to 1300 °C).

### 3.2. Desorption in Distilled Water

The sorbent granules after contact with the model solution were tested in distilled water to investigate the desorption. The results of desorption tests in distilled water after 30 days of exposure are provided in Table 2.

Analyzing the data provided in Table 2, it can be seen that no desorption was observed after the 30 days of exposure, except the change in Ca^2+^ ion content and pH.

### 3.3. Phase Composition

**Figure 4:**
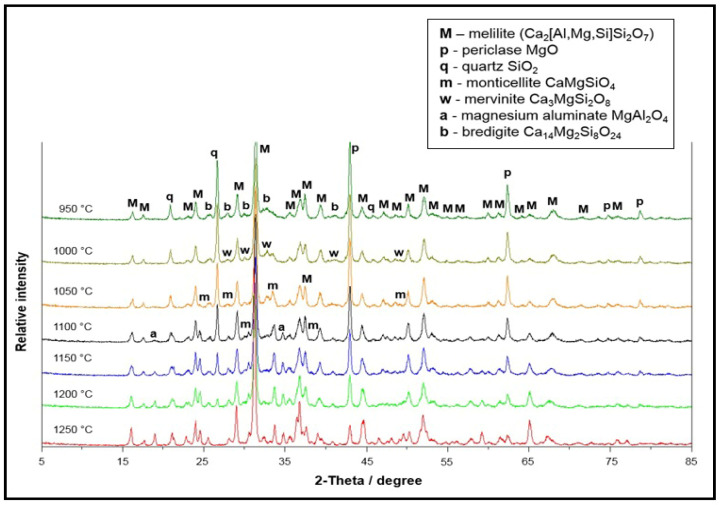
XRD phase compositions of sorbent depending on sintering temperature: M—melilite (Ca_2_[Al,Mg,Si]Si_2_O_7_); p—periclase MgO, q—quartz SiO_2_, m—monticellite CaMgSiO_4_, w—mervinite Ca_3_MgSi_2_O_8_, a—magnesium aluminate MgAl_2_O_4_, b—bredigite Ca_14_Mg_2_Si_8_O_24_.

During preparation, an organometallic compound based on esters was added to the mixture as a modifier, which was simultaneously used as a temporary binder and a source of inovalent ions. During further heat treatment, the inovalent ions participated in reactions of defect formation and activated sintering Ca^2+^ ↔ 2Na^+^+p with subsequent heating. The phase compositions of sorbent, depending on sintering temperature, are shown in Figure 4.

**Figure 5:**
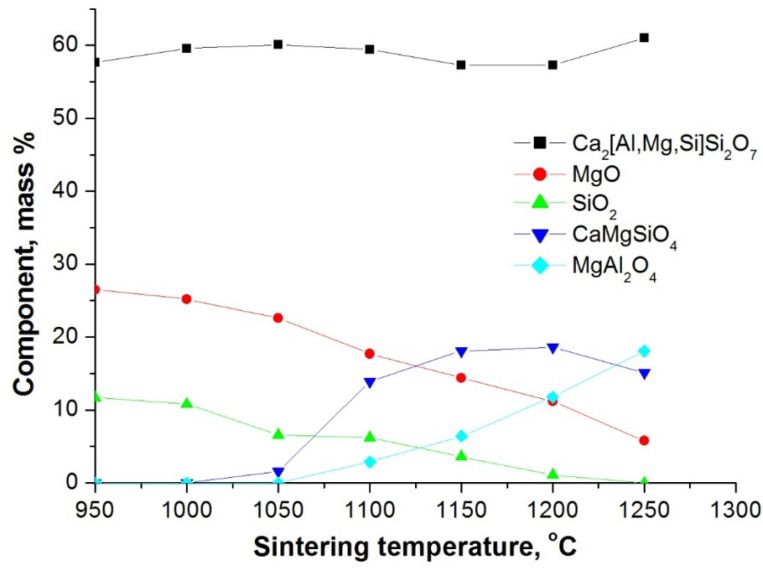
Sequence of the phase formation process during sorbent synthesis depending on sintering temperature derived from XRD patterns.

The sequence of the phase formation process in the temperature range of formation of sorbent granules, compiled according to the results of XRD analysis, are provided in Figure 5.

It is seen from XRD patterns that the main phase component is melilite (Ca_2_[Al,Mg,Si]Si_2_O_7_) forming in a wide temperature range. The phases mervinite and bredigite were neglected in Figure 5, as both are in the general melilite group Ca_2_[Al,Mg,Si]Si_2_O_7_. The temperature range corresponding to the decrease in MgO and SiO_2_ content correlates with the temperature interval of monticellite (CaMgSiO_4_) formation. Binding of free MgO also occurs with the formation of aluminomagnesium spinel (MgAlO_4_).

**Figure 6:**
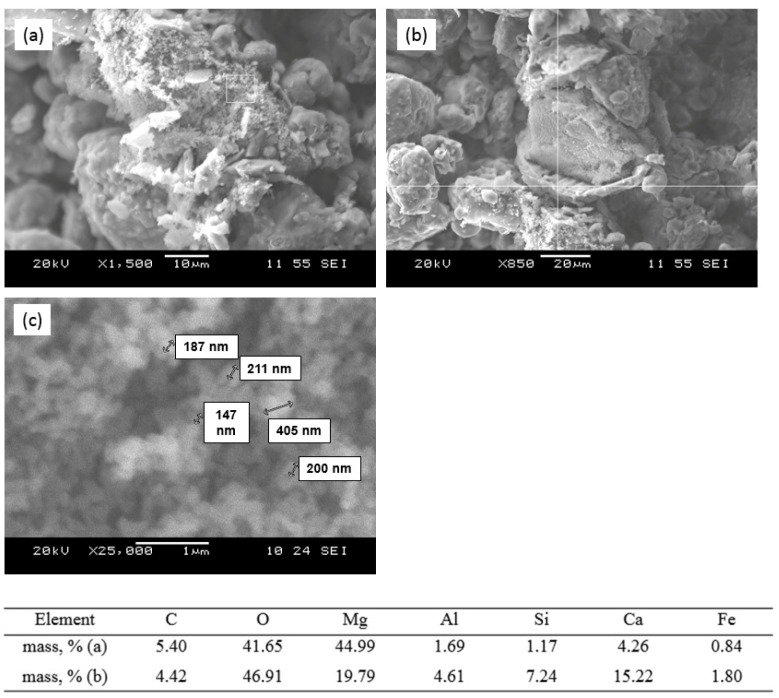
SEM images and analysis of the sorbent surface after sintering at a temperature of 900 °C: (**a**) MgO in the volume of original decomposing calcium–magnesium carbonate grain; (**b**) phase formation of the mixed calcium–magnesium aluminosilicate (melilite) on the periphery of the decomposing carbonate grain; (**c**) particle nanosize of the newly formed MgO.

**Figure 7:**
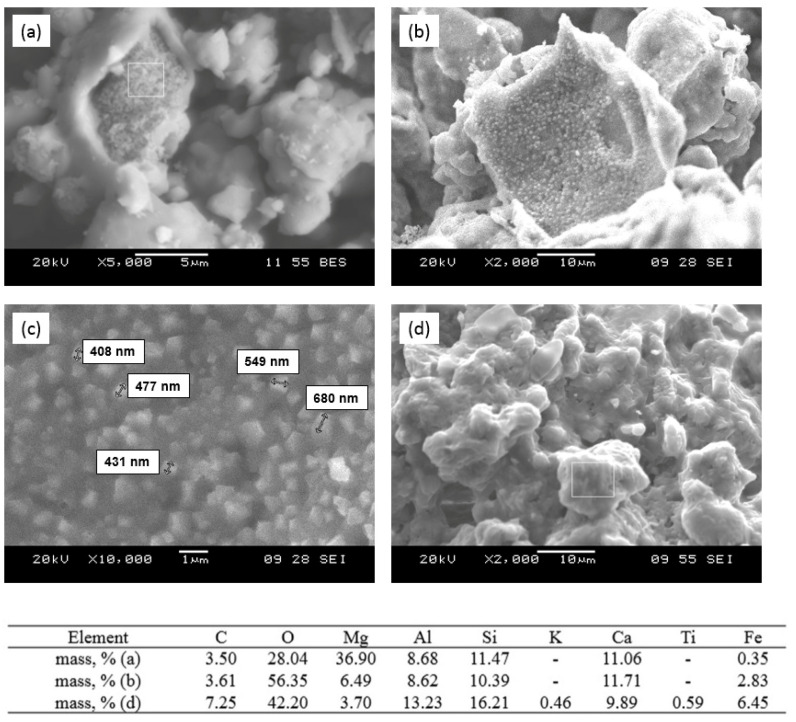
SEM images and analysis of the sorbent surface after sintering: (**a**) phase formation of calcium and magnesium aluminosilicates in the volume of decomposing carbonate grains at a temperature of 1050 °C; (**b**) binding of MgO at a temperature of 1150 °C; (**c**) nanosized mixed aluminosilicates formed in the volume of decomposing carbonate grains at a temperature of 1150 °C; (**d**) melting of iron-enriched particles of the newly formed mixed aluminosilicates at a temperature of 1250 °C.

According to the results of electron microscope analysis, the process of phase formation of the sorbent components passes through the following three main stages:

1. Decomposition of the initial grains of calcium–magnesium carbonate with the formation of MgO and mixed calcium and magnesium aluminosilicates at the grain periphery (Figure 6). Formation of the nanostructured MgO in the volume of decomposing grains at temperatures from 850 to 950 °C.

2. Expansion of the phase formation reaction zone of mixed calcium–magnesium aluminosilicates in the volume of decomposing calcium–magnesium carbonate grains, decomposition of carbonate grains (Figure 7a) and formation of nanosized calcium–magnesium aluminosilicates (Figure 7c) in the temperature range of 1000–1150 °C, corresponding in composition to melilite.

3. Glass formation due to partial melting of the newly formed particles (Figure 7d) in the temperature range of 1200–1350 °C.

**Figure 8:**
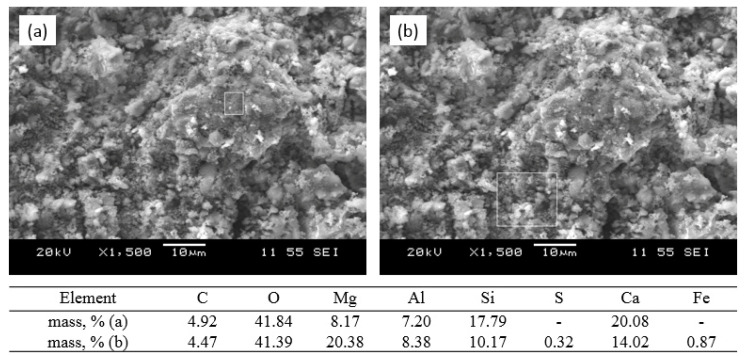
SEM images and analysis of the sorbent surface in different areas (**a**,**b**) after sintering at a temperature of 1100 °C.

Thereby, the formation of aluminosilicate structure of material during sintering almost completely proceeds in the temperature range of 1100–1150 °C. The chemical composition of the surface is variable in different areas (Figure 8a,b), which corresponds to the structure of melilite within homogeneity of the solid solution. It should be emphasized that the residual carbon remains in the composition of the sintered material.

### 3.4. Sorption–Mineralization Mechanism

The nature of physical and chemical processes occurring in the sorbent–sorbate system was clarified by comparing the results of sorption tests with electron microscope analysis of the sorbent surface after interaction with the copper sulfate solution. The first interval of sintering temperature I in the range of 850 to 1000 °C can be characterized as a precipitation interval, which is associated with the presence of the free oxides MgO and CaO in the sorbent structure, which are formed during decomposition of the initial carbonates (Figure 6). The highly active structural state of decomposition products and incomplete phase formation of the aluminosilicate matrix enable the migration of Mg^2+^ and Ca^2+^ ions to the sorbate solution and the neutralization reaction of the acidic copper sulfate solution with homogenous precipitation of the interaction products in the solution volume.

With the increase in sintering temperature from 1000 to 1200 °C (Figure 7) of sorbent-mineralizer, and the formation of aluminosilicates of melilite series in its structure, the homogeneous precipitation stage is suppressed and the topochemical processes are activated. The topochemical character of the sorption process from sorbate solution was followed by the decrease in Cu^2+^ ion concentration in the solution volume by between 92% and 95%, with an absence of precipitation and an increase in the sorbent’s mass by 0.6%.

The sintering temperature of 1200 °C was critical, as the sorption properties were lost with further sintering temperature increase. The latter was connected with partial smelting and deactivation of the sorbent–mineralizer surface (Figure 7d). Thereby, an optimum temperature interval for the formation of the structure and properties of sorbent-mineralizer is the temperature range from 1000 to 1150 °C.

**Figure 9:**
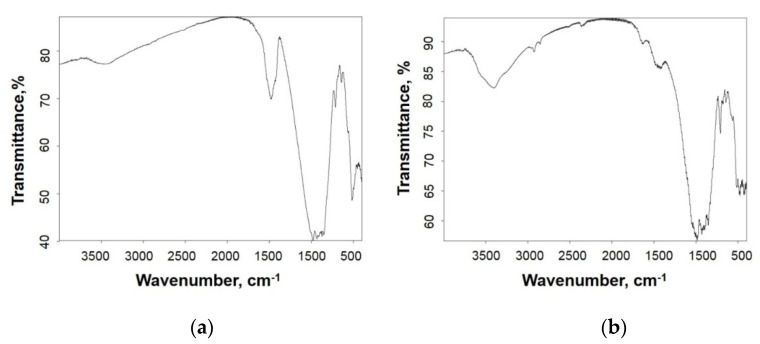
Infrared spectrum for: (**a**) original sorbent and (**b**) after interaction with the CuSO_4_ model solution, sintering temperature 1050 °C.

The nature of topochemical processes at the sorbent–sorbate interface was clarified by studying the structure of the short-range order of the sorbent after interaction with the copper sulfate solution. The peculiarities of the short-range order of the sorbent are illustrated by the results of infrared spectroscopic analysis (Figure 9a,b). The absorption band in the frequency range of 900 to 1000 cm^−1^ corresponds to the vibrations of the Si-O bond of the SiO_4_ tetrahedron. The multiplet is due to the influence of Al^3+^ and Ca^2+^ ions on the power characteristics of the Si-O bond, which causes a shift in the electron density [40,41].

The 1450 cm^−1^ absorption band corresponds to the valence vibrations of the C-O bond. The absence of the 850 cm^−1^ band corresponding to the deformation vibrations of the C-O bond in CO_3_^2−^ confirms that the CO_3_^2−^ groups are structurally connected with silicate and have a deformation effect on the Si-O bonds of the SiO_4_ tetrahedron. Similarly, the absorption band in the region of 3450 cm^−1^ corresponding to the valent vibrations of O-H should be attributed to the vibrations of the free OH^−^ groups, since the band 1650 cm^−1^ associated with deformation vibrations of OH^−^ groups in the H_2_O molecule is absent [42]. It means that both CO_3_^2−^ and OH^−^ groups are structurally connected with the silicate matrix and represent the primary active centers.

**Figure 10:**
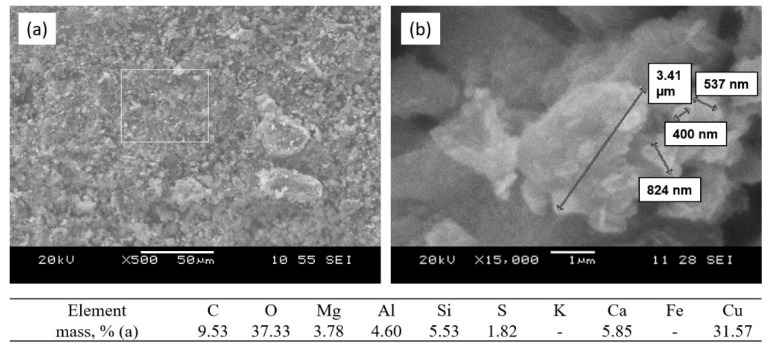
SEM images and analysis of the sorbent surface after interaction with the CuSO_4_ model solution: (**a**) melilite surface after Cu^2+^ ion sorption; (**b**) mixed nanostructured calcium–magnesium aluminosilicates; sintering temperature 1050 °C.

After interaction of the sorbent with copper sulfate solution, the deformation of the SiO_4_ tetrahedron is characterized by broadening and multipletting of the 900 to 1000 cm^−1^ band. The additional deformation of the SiO_4_ tetrahedron can be explained by the appearance of Si–O–Cu bonds in the short-range order structure, along with Si–O–Mg and Si–O–Ca bonds, i.e., by the partial replacement of Ca^2+^ and Mg^2+^ by Cu^2+^ in the aluminosilicate structure. In turn, the CO_3_^2−^ and OH^−^ groups bound to the aluminosilicate can serve as active centers of the topochemical phase formation of mixed carbonate structures. Thereby, the possibility of Ca^2+^ ↔ Cu^2+^ and Mg^2+^ ↔ Cu^2+^ ion exchange reactions and topochemical phase formation on the sorbent-mineralizer surface is confirmed by the results of IR spectroscopic analysis. The results of the surface study of the new formations by SEM analysis demonstrate the versatility of physicochemical processes of interaction and phase formation in the MgO-CaO-Al_2_O_3_-SiO_2_-CO_2_ system (Figure 10a,b).

**Figure 11:**
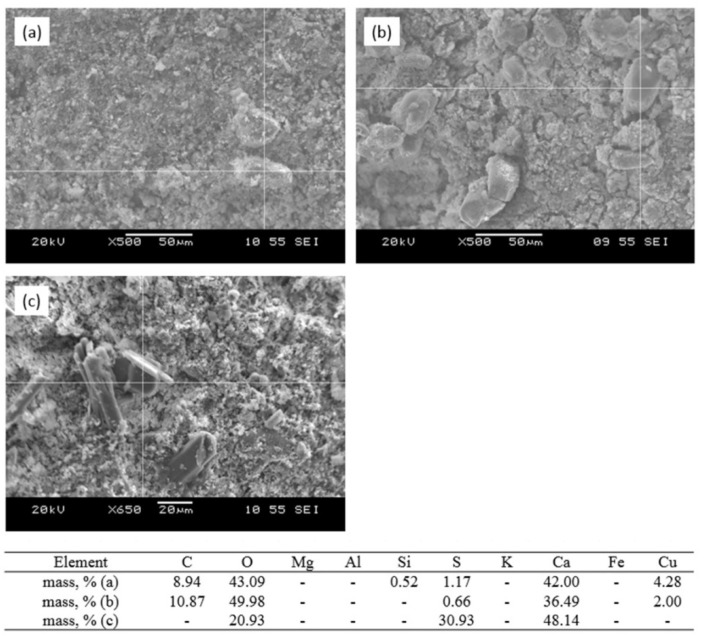
SEM images and analysis of the sorbent surface after interaction with the CuSO_4_ model solution: (**a**) mixed calcium–copper sulfocarbonate; (**b**) mixed calcium–copper carbonate; (**c**) surface growth of the calcium-sulfate formations, sintering temperature 1050 °C.

The following three stages of the process can be distinguished:

- sorption of Cu^2+^ ions by melilite as a result of ion exchange reactions with formation of the new nanosized formations of the mixed aluminosilicates (Figure 10a,b);
- extraction of the ion exchange products;
- surface phase formation as mixed sulfocarbonates (Figure 11a), carbonates (Figure 11b), and sulfates (Figure 11c) of calcium and copper, which are structurally connected with aluminosilicate matrix.

Summarizing the above-mentioned aspects, the ion exchange reactions are elementary acts of interaction and phase formation in the MgO-CaO-Al_2_O_3_-SiO_2_-CO_2_ system, which determine the sorption–mineralization capability of the sorbent.

### 3.5. Comparison of Various Cu^2+^ Sorbents

The sustainable granular composite of MgO-CaO-Al_2_O_3_-SiO_2_-CO_2,_ and the results of the sorption tests were compared with the other sorbents available in literature, as listed in Table 3.

Analyzing the data provided in Table 3, it can be seen that the direct comparison of the performances of the different sorbents is complicated due to various experimental conditions. In the present work, the sorption tests were static, whereas a number of the sorption studies available in literature were performed under dynamic conditions. It can be speculated that the granular composite of MgO-CaO-Al_2_O_3_-SiO_2_-CO_2_ in the present work has similar performances as multiwalled carbon nanotubes or activated carbon. Magnetic porous ferrospinel MnFe_2_O_4_ [43], graphene oxide [20] and carbonaceous sulfur-containing chitosan–Fe(III) [18] seem to be superior. However, during comparison it was also necessary to take into account the sorption irreversibility; the novel granular composite is characterized by the irreversibility of the sorption process and mineralizing properties, whereas the magnetic porous ferrospinel MnFe_2_O_4_ [43] is reversible and can be reused a few times.

## 4. Conclusions

The present study investigated obtaining mineralizing sorbent based on MgO-CaO-Al_2_O_3_-SiO_2_-CO_2_ system with specified composition and properties. The irreversible sorption of the Cu^2+^ ions occurred by the ion exchange reaction of Ca^2+^ ↔ Cu^2+^ and Mg^2+^ ↔ Cu^2+^ and by introduction into melilite solid solutions. The primary active nanosized adsorption centers on the sorbent-mineralizer surface participated in the ion exchange reactions 2OH^−^→CO_3_^2−^, 2OH^−^→SO_4_^2−^ and CO_3_^2−^ → SO_4_^2−^ and surface phase formation, obtaining new mixed carbonate and sulfate structures connected with the silicate matrix. The processes of sorption and surface phase formation were parallel and did not interfere with each other, forming sorption–mineralizing properties that excluded migration of ions to the environment. In the sorption stage of the process, the ions of Mg^2+^ mainly participated in the ion exchange reactions. The irreversibility of the sorption process was confirmed by desorption tests in distilled water.

In conclusion, a mixture of natural calcium carbonates, magnesium carbonates and aluminosilicate clay materials with binder additions can be used for sorbent obtaining and for subsequent purification of large natural water bodies contaminated by Cu^2+^ ions, by placing the sorbent at the bottom of the water bodies, or using barrier technology. The irreversibility of the process and the mineralization enable permanent storage of the sorbents at the bottom of the water bodies, preventing copper pollution.

## Figures and Tables

**Table 1:** XRF analysis of initial sorbent mixture.

Compound	Content, Mass %	Compound	Content, Mass %
Al_2_O_3_	14.70	MnO	0.03
CaO	28.20	NiO	0.04
Cr_2_O_3_	0.05	P_2_O_5_	0.10
CuO	0.06	SiO_2_	25.38
Fe_2_O_3_	1.49	TiO_2_	0.86
K_2_O	0.12	ZnO	0.01
MgO	23.36	LOI	0.20

**Table 2:** Results of the desorption tests in distilled water after 30 days of exposure (except the change in Ca^2+^ ions content and pH).

Element	Content before Exposure, mg/L	Content after 30 Days Exposure Time, mg/L
Al	0	0
Ca	0	27.2840
Si	0	0
Na	0	0
K	0	0
Cu	0	0
pH		8.31

**Table 3:** Comparison of Cu^2+^ sorbents.

Type of Sorbent	Initial Concentration, mg/L	Intake of Cu^2+^, mg/g	Contact Time (Test Type), h	
MgO-CaO-Al_2_O_3_-SiO_2_-CO_2_ system	500	16.7	168 (static)	This study
Graphene oxide	15	75	24 (dynamic)	[20]
Multiwalled carbon	15	2	24 (dynamic)	[20]
nanotubes				
Activated carbon	15	14.7	24 (dynamic)	[20]
Magnetic porous ferrospinel MnFe_2_O_4_	–	60.5	3 (dynamic)	[43]
Carbonaceous sulfur-containing chitosan–Fe(III)	500	413.2	0.25 (dynamic)	[18]

## Data Availability

The data presented in this study are available on request from the corresponding authors.
