# Correction: Morozova et al. Insights into Sorption–Mineralization Mechanism for Sustainable Granular Composite of MgO-CaO-Al_2_O_3_-SiO_2_-CO_2_ Based on Nanosized Adsorption Centers and Its Effect on Aqueous Cu(II) Removal. *Nanomaterials* 2022, *12*, 116

**DOI:** 10.3390/nano16020119

**Published:** 2026-01-16

**Authors:** Alla G. Morozova, Tatiana M. Lonzinger, Vadim A. Skotnikov, Gennady G. Mikhailov, Yury Kapelyushin, Mayeen Uddin Khandaker, Amal Alqahtani, D. A. Bradley, M. I. Sayyed, Daria I. Tishkevich, Denis A. Vinnik, Alex V. Trukhanov

**Affiliations:** 1 Laboratory of Single Crystal Growth, South Ural State University, 454080 Chelyabinsk, Russia; morozovaag@susu.ru (A.G.M.); lonzingertm@susu.ru (T.M.L.); skotnikovva@susu.ru (V.A.S.); mikhailovgg@susu.ru (G.G.M.); kapeliushinye@susu.ru (Y.K.); dashachushkova@gmail.com (D.I.T.); denisvinnik@gmail.com (D.A.V.); 2 Centre for Applied Physics and Radiation Technologies, School of Engineering and Technology, Sunway University, Petaling Jaya 47500, Selangor, Malaysia; mayeenk@sunway.edu.my (M.U.K.); d.a.bradley@surrey.ac.uk (D.A.B.); 3 Department of Basic Sciences, Deanship of Preparatory Year and Supporting Studies, Imam Abdulrahman Bin Faisal University, Dammam 34212, Saudi Arabia; amalqahtani@iau.edu.sa; 4 Centre for Nuclear and Radiation Physics, Department of Physics, University of Surrey, Guildford GU2 7XH, UK; 5 Department of Physics, Faculty of Science, Isra University, Amman 11622, Jordan; dr.mabualssayed@gmail.com; 6 Department of Nuclear Medicine Research, Institute for Research and Medical Consultations (IRMC), Imam Abdulrahman Bin Faisal University, Dammam 31441, Saudi Arabia; 7 Laboratory of Magnetic Films Physics, SSPA “Scientific-Practical Materials Research Centre of NAS of Belarus”, 220072 Minsk, Belarus

**Error in Figure**

In the original publication [1], there was a mistake in Figure 10b as published. The caption of Figure 10a has also been updated. The corrected Figure 10 appears below.

**References**

There were three citation mistakes in the main text.

In Section 2.2. Sintering, the references 34,35 should be updated as 35,36; the reference 36 should be updated as 37; the references 37,38,39,40,41,42 should be updated as 38,39.

In Section 3.4. Sorption–Mineralization Mechanism, the references 31,32 should be updated as 40,41; the reference 35 should be updated as 42.

In Section 3.5. Comparison of Various Cu^2+^ Sorbents, the references 34 and 38 should be updated as 43.

The authors state that the scientific conclusions are unaffected. This correction was approved by the Academic Editor. The original publication has also been updated.

## Figures and Tables

**Figure 10:**
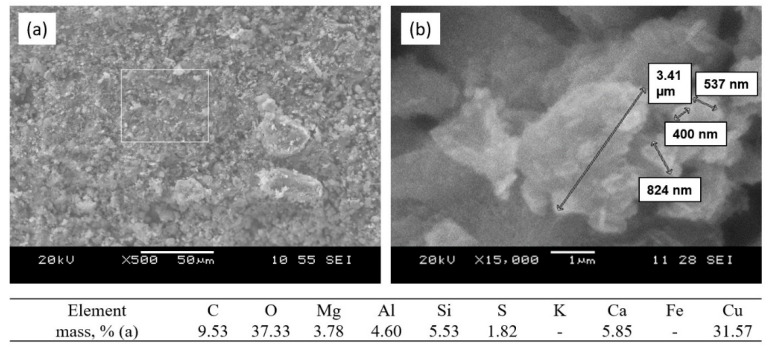
SEM images and analysis of the sorbent surface after interaction with the CuSO_4_ model solution: (**a**) melilite surface after Cu^2+^ ion sorption; (**b**) mixed nanostructured calcium–magnesium aluminosilicates; sintering temperature 1050 °C.
